# Cellulose Nanofibers Isolated from the *Cuscuta Reflexa* Plant as a Green Reinforcement of Natural Rubber

**DOI:** 10.3390/polym12040814

**Published:** 2020-04-04

**Authors:** Midhun Dominic C.D., Rani Joseph, P.M. Sabura Begum, Meera Joseph, Dileep Padmanabhan, Leonna Angela Morris, Athira S Kumar, Krzysztof Formela

**Affiliations:** 1 Department of Chemistry, Sacred Heart College (Autonomous), Kochi, Kerala PIN-682013, India; meerajoseph1995@gmail.com (M.J.); leonnamorris19@gmail.com (L.A.M.); 2 Department of Applied Chemistry, Cochin University of Science and Technology (CUSAT), Kerala PIN-682022, India; pmsabura@cusat.ac.in; 3 Department of Polymer Science and Rubber Technology, Cochin University of Science and Technology (CUSAT), Kerala PIN-682022, India; rani@cusat.ac.in (R.J.); pdileep84@gmail.com (D.P.); 4 St. Albert’s College (Autonomous), Kochi, Kerala PIN-682018, India; athirask25@gmail.com; 5 Department of Polymer Technology, Faculty of Chemistry, Gdańsk University of Technology, Gabriela Narutowicza 11/12, 80–233 Gdańsk, Poland

**Keywords:** natural rubber, cellulose nanofibers, *Cuscuta reflexa* plant, reinforcement, matrix-filler interactions

## Abstract

In the present work, we used the steam explosion method for the isolation of cellulose nanofiber (CNF) from *Cuscuta reflexa*, a parasitic plant commonly seen in Kerala and we evaluated its reinforcing efficiency in natural rubber (NR). Fourier Transform Infrared Spectroscopy (FTIR), X-Ray Diffraction (XRD), Scanning Electron Microscopy (SEM), Transmission Electron Microscopy (TEM), and Thermogravimetric analysis (TGA) techniques indicated that type I cellulose nanofibers, with diameter: 10–30 nm and a 67% crystallinity index were obtained by the proposed method. The results showed that application of CNF in NR based nanocomposites resulted in significant improvement of their processing and performance properties. It was observed that the tensile strength and tear strength of NR/CNF nanocomposites are found to be a maximum at 2 phr CNF loading, which corresponds with the studies of equilibrium swelling behavior. Dynamic mechanical analysis, thermogravimetric analysis, and morphological studies of tensile fractured samples also confirm that CNF isolated from *Cuscuta reflexa* plant can be considered as a promising green reinforcement for rubbers.

## 1. Introduction

Carbon black, precipitated silica, clay, and calcium carbonate are generally used as fillers in the rubber industry. Recently, attempts are being made to limit the application of carbon black in rubber compounding because of its high cost, non-biodegradable nature, non-renewable origin, and the pollution hazards. Several works were reported in the literature for preparing eco-friendly composites by using green-fillers like rice husk ash [1], marble sludge [2], natural fiber [3], bagasse ash [4], chitin [5], eggshell powder [6], etc. Among these green-fillers, utilization of natural fibers as reinforcing filler in rubber is a very active area because of their unique properties like biodegradability, recyclability, high aspect ratio, less toxicity, high modulus, low cost, etc. Natural-fiber reinforced composites have already made a significant presence in various sectors such as automotive, electrical and electronics, sports goods, building/construction, packaging, adhesives, etc.

Lignocellulosic fibers from jute, sisal, isora, coir, wheat bran, pineapple leaf, silk waste, bamboo, agave, kenaf, oil palm, hemp, and banana have already been used as reinforcements in different matrices [7]. Due to the lesser affinity between hydrophilic fiber and the hydrophobic natural rubber matrix, the tensile strength of untreated fiber reinforced natural rubber composites show a deteriorating effect compared to neat polymer. The tensile properties of natural rubber can be significantly improved by the proper surface modification of either the matrix or the reinforcement phase. The tensile properties and abrasion resistance of natural rubber were improved by the utilization of short *Caryota* fibers of size 10 mm by latex stage processing using yeast as coagulating agent [8]. Studies show that the alkali-treated oil palm fiber reinforced natural rubber has got better mechanical and technological properties compared to untreated fiber-NR vulcanizates [9]. Sustainable rubber composites can be prepared by using short aramid fiber-carbon black hybrid filler [10]. The swelling behavior and transport of solvents in fiber-reinforced composites were also well established. The organic solvent uptake of natural rubber was tailored by using bamboo pulp derived cellulose nanofibers and nanowhiskers [11]. The synergistic use of short cellulose fibers and bis(triethoxysilylpropyl)tetrasulphide modified silica offer greater processibility and lower environmental impact compared to conventional silica-filled rubber composite [12].

A recent study reported in the literature shows that wheat bran can be used as an environment friendly replacement for commercial cellulose fillers [13]. It shows that Wolff activity coefficient was positive for the composites prepared from wheat bran and natural rubber, whereas the tensile strength and elongation at break show a decreasing trend. The dynamic mechanical analysis of treated-oil palm-fiber filled natural rubber vulcanizates shows an improvement in storage modulus and glass transition temperature compared to the untreated fiber-filled composites [3]. The pronounced improvement in mechanical properties was obtained in the longitudinal direction than in the transverse direction in the case of short isora fibers (isolated from *Helicteres isora*) filled vulcanizates with resorcinol formaldehyde as the bonding agent [14]. The homogenous dispersion of the fiber, the fiber orientation, and the matrix-fiber interphase play a key role in enhancing the mechanical properties of the composites. The chemical modification methods like, silylation, mercerization, acetylation, maleated coupling, and enzyme treatment are usually adopted to upgrade the interaction between the fiber and the matrix [3].

It was observed that 3,3’-dithiopropionic acid chloride (DTACl) modified cellulosic nanocrystals (CNC) can covalently bound to styrene–-butadiene rubber (SBR) effectively [15]. The homogeneous dispersion of DTACl modified cellulose nanocrystals provides an additional strain stiffening effect which was similar to the behavior exhibited by silica and carbon black in elastomers. The mercapto-modified cellulose nanocrystals in natural rubber show improved thermo-mechanical properties and reduced stress softening (Mullins effect) [16]. A recent study was conducted to replace carbon black with rice husk derived nanocellulose in natural rubber for green tire manufacturing [17]. The authors have reported that a significant reduction in rolling resistance was possible while replacing 50 phr carbon black with 45 phr carbon black and 5 phr nanocellulose in natural rubber vulcanizates. The tensile storage modulus of natural rubber at 25 °C was significantly improved by the addition of 2.5 wt % cellulose nanocrystals derived from soy hulls [18]. Tremendous improvement in mechanical properties and conductivity were observed when polyaniline coated cellulose nanofibers were introduced into natural rubber [19].

In this study, *Cuscuta reflexa* plant is proposed to be used as the source for the extraction of cellulose nanofibrils. *Cuscuta reflexa* commonly called dodder plant belongs to the *Cuscutaceae* family, commonly seen in Kerala. It is a stem holoparasite. The plant is leafless, rootless, and it has no chlorophyll content. Agriculturalists consider *Cuscuta reflexa* as a destructive weed because it infests most dicotyledonous plants [20]. Alkali treatment of the raw *Cuscuta reflexa* followed by the steam explosion in the presence of oxalic acid is proposed to be used for the extraction of cellulose nanofiber (CNF). The acid hydrolysis is likely to induce defibrillation and breakdown of polysaccharides into simple sugars thereby facilitating the production of *Cuscuta reflexa* nanofibers [21]. CNF from *Cuscuta reflexa* are proposed to be dispersed in NR latex by preparing masterbatches. Then the masterbatch is proposed to be processed with conventional vulcanizing agents in a two-roll mill to get the desired CNF content in the composite. The prepared NR–CNF nanocomposites were analyzed for different properties. Thus the prescribed work has mainly two advantages. Firstly, it utilizes the most dangerous parasitic plant in India for the preparation of a value-added product, CNFs, thereby diminishing the threat caused by this plant. As far as we know, no attempts have been put forward so far to evaluate the use of CNF isolated from *Cuscuta reflexa* as reinforcement in elastomers. Secondly, natural rubber based green composites with good mechanical, technological, dynamic mechanical, thermal, and barrier properties were prepared using CNF without using any costly coupling agents.

## 2. Materials and Methods 

### 2.1. Materials

*Cuscuta reflexa,* the parasitic plant was collected from the premises of Thevara, Kochi. Centrifuged NR latex concentrate with a minimum 60% dry rubber content (DRC), preserved with high ammonia (HA) preservative system and conforming to the BIS 5430-1981 specifications was used in the study. Natural rubber (ISNR-5) was supplied by the Rubber Research Institute of India. Zinc oxide was supplied by M/s Meta Zinc Ltd., Mumbai, India. Stearic acid was obtained from Godrej Soaps Pvt. Ltd., Mumbai, India. N-cyclohexyl-2-benzothiazole sulfenamide (CBS) was obtained from Merchem Ltd., Cochin, India. Sulphur was obtained from Standard Chemicals Co. Pvt. Ltd., Chennai, India. Styrenated phenol (Nonox SP) used in this study was obtained from Olympic Chemicals, Maharashtra, India. NaOH, oxalic acid and acetic acid were supplied by Merck Specialities Pvt. Ltd., Mumbai, India. 

### 2.2. Methods

#### 2.2.1. Preparation of Cellulosic Nanofibers

*Cuscuta reflexa* fibers were collected, washed thoroughly and cut into small pieces of approximate size 1 cm. About 100 g of raw *Cuscuta reflexa* fibers were treated with 2% NaOH solution at 60 °C for 1 h (fiber to liquid solution ratio 1:10 g/mL). Alkali treatment was done to remove a major part of lignin and hemicellulose from raw *Cuscuta reflexa* fiber. It was washed well to carry away the alkali content and subjected to the steam explosion with 5% oxalic acid in an autoclave until it attained a pressure of 20 psi. The pressure was released immediately. The autoclave was again set to reach a pressure of 20 psi, and the *Cuscuta reflexa* fibers were kept under that pressure for 15 min. The pressure was released and the process was repeated 3 times. The fibers were taken out, washed well to get rid of acid. 

#### 2.2.2. Preparation of NR–CNF Nanocomposites

NR–CNF composites were prepared in a two-step process. First step involves the masterbatch preparation in NR latex followed by compounding the masterbatch with solid NR and vulcanizing agents using a two-roll mill. A good dispersion of CNF in NR latex (60% DRC) was obtained by mixing the requisite amount of CNF in NR latex for 3 h in the presence of stabilizers. Dilute acetic acid was added to coagulate the latex. The coagulum was squeezed between the rollers to remove water. The obtained sheet was oven dried at 60 °C. CNF loadings were adjusted to get 1, 2, 3, and 4 phr fiber in the final composites. In the second step, the compounding of the masterbatch with solid NR and other vulcanizing agents was carried out using a two-roll mill. Fibers were oriented in the mill direction by passing through the tight gap in the mill at the end of the mixing process. The formulation for preparing NR–CNF composites is given in Table 1. Vulcanization of various test samples was carried out in an electrically heated hydraulic press having 45 cm × 45 cm platen at 150 °C at a pressure of 2844 psi on the mold up to optimum cure time. Molded samples were conditioned for 24 h.

#### 2.2.3. Characterization Techniques

The chemical composition of nanofibers at each stage of treatment was determined according to the ASTM standards cellulose (ASTM D 1103-55T), hemicellulose (ASTM D 1104-56), and lignin (ASTM D 1106-56). The cure characteristics were determined by using Rubber Process Analyzer, RPA 2000 as per ASTM D 5289. Stress–strain tests were carried on a Shimadzu Model AGI Universal Testing Machine as per ASTM D 412. Tear resistance of the samples was tested as per ASTM D 624. The hardness of samples was measured as per ASTM D 2240 by shore A type Durometer. The abrasion resistance was performed using Bariess DIN abrader, Oberdischingen, Germany (ASTM D 5963). Compression set was studied on a compression set apparatus as per ASTM D 395 with a spacer thickness 9.5 mm. Rebound resilience of NR–CNF nanocomposites was determined by vertical rebound method according to ASTM D 2832-88. Fourier transform infrared analysis was conducted on Thermo Nicolet, Avatar 370 model IR spectrometer, Waltham, MA, USA, in 4000–400 cm^−1^ spectral range with a resolution of 4 cm^−1^. Thermogravimetric analysis was performed on thermogravimetric analyzer Q-50, TA instruments with a heating rate of 20 °C/min under nitrogen atmosphere. The fracture surfaces of the vulcanized samples were studied with JOEL JSM 8390 LV scanning microscope (JEOL USA, Inc., Peabody, MA, USA). Transmission Electron Microscopy (TEM) imaging was done using JEOL JEM 2100 High resolution transmission electron microscope (JEOL USA, Inc., Peabody, MA, USA). X-Ray Diffraction (XRD) analysis was conducted using Bruker AXS D8 Advance X-Ray powder diffractometer (Bruker, MA, USA). The crystallinity index ($I_{C}$) of raw *Cuscuta* fiber, alkali-treated fiber and CNF was calculated using Equation (1) [22]:

(1) $$I_{C}\left(\%\right)=\left(1-\frac{I_{am}}{I_{002}}\right)\times 100\%$$

where $I_{002}$ is the maximum intensity of diffraction of the (002) lattice peak (22°–23°), and $I_{am}$ is that of the amorphous material between 18° and 19° where the intensity is minimum [23]. 

Swelling behavior and cross-link density of NR–CNF nanocomposites were determined by equilibrium swelling method (samples kept at room temperature for 3 days). For NR based nanocomposites, toluene was used as the solvent. Circular samples were punched out from vulcanized sheets using a sharp-edged steel die. The samples were weighed and dipped in 30 mL solvent in diffusion bottles. Samples were taken out at regular intervals of time, wiped of the excess solvent and weighed. Each time after weighing they were immediately immersed into the bottles and weighing continued until they reached equilibrium swelling. Finally, the deswollen weight of the samples was taken after drying. Mol % uptake (*Q*_t_) of solvent for the samples was calculated using Equation (2):

(2) $$Q_{t}=\left(\frac{(W_{S}-W_{i})/M_{s}}{W_{i}}\right)\times 100\%$$

where *W*_s_ is the weight of the swollen sample and *W*_i_ the initial weight of the sample i.e., the weight of the sample before swelling, *M*_s_ the molar mass of solvent.

Swelling index was calculated using the following Equation (3):

(3) $$Swelling\;index=\frac{W_{s}-W_{i}}{W_{i}}$$

where *W*_s_ is the weight of the swollen sample and *W*_i_ the initial weight of the sample i.e., the weight of the sample before swelling.

Cross-link density (ν) was calculated according to Equation (4):

(4) $$\nu =\frac{1}{2M_{c}}$$

where *M*_c_ is the molecular mass between successive cross-links. *M*_c_ is given by the Flory–Rehner Equation (5) [24]:

(5) $$M_{c}=\frac{-\rho_{r}V_{s}V_{r}^{1/3}}{\ln\left(1-V_{r}\right)+V_{r}+\chi V_{r}^{2}}$$

where *ρ*_r_ is the density of rubber, *V*_s_ the molar volume of the solvent, *χ* the rubber solvent interaction parameter and *V*_r_ the volume fraction of swollen rubber which was calculated using Ellis and Welding Equation (6) [25]:

(6) $$V_{r}=\frac{\left(d-fw\right)\rho_{r}^{-1}}{\left(d-fw\right)\rho_{r}^{-1}+A_{s}\rho_{s}^{-1}}$$

where *d* is the deswollen weight of the polymer, *f* the volume fraction of insoluble components, *w* is the initial weight of the polymer, A_s_ the amount of solvent absorbed and *ρ*_s_ density of the solvent. *χ* is determined using Hildebrand Equation (7):

(7) $$\chi =\beta +\frac{V_{s}{\left(\delta_{s}-\delta_{p}\right)}^{2}}{RT}$$

where β e is the lattice constant, *R* the universal gas constant, *T* the absolute temperature, *δ*_s_ and *δ*_p_ are solubility parameters of the solvent and polymer, respectively. The elastic Gibbs free energy was calculated using Flory–Huggins Equation (8):

(8) $$\Delta G=RT\left[\ln\left(1-V_{r}\right)+V_{r}+\chi V_{r}^{2}\right]$$

TA Instruments DMA Q800 Dynamic mechanical analyzer (TA Instruments Inc., New Castle, DE, USA) was used to measure dynamic mechanical properties of the samples in tension mode. Samples for DMA measurement were cut from the vulcanized sheets, having the dimension 30 mm × 3 mm × 2 mm. For the temperature sweep analysis, the samples were tested from −80 to 80 °C at a ramp rate of 3 °C/min and a frequency of 1 Hz.

The effectiveness of CNF on the storage moduli of the composites can be represented by a coefficient *C* which is given by Equation (9):

(9) C=(E′gE′r)composite(E′gE′r)matrix 

where ${E^{\prime }}_{g}$ and $E^{\prime }{}_{r}$ are storage modulus in the glassy and rubbery region, respectively.

The amount of polymer chains immobilized through matrix-filler interactions is determined by using Equation (10) [13]:

(10) $$C_{v}=1-\left(1-C_{0}\right)\frac{W}{W_{0}}$$

where $C_{v}$ is the volume fraction of the immobilized polymer chains, $C_{0}$ stands for the volume fraction of the immobilized polymer chains in the pure natural rubber (taken to be zero), and W and $W_{0}$ are energy loss fraction for NR–CNF composite and pure NR, respectively. Energy loss fractions *W* can be calculated from the tan δ in accordance to Equation (11):

(11) $$W=\frac{\pi \tan\delta }{\pi \tan\delta +1}$$

For better understanding the interfacial interactions between NR and used CNF isolated from *Cuscuta reflexa*, the adhesion factor (*A*) was calculated from the loss factor (tanδ) using Equation (12) [13]:

(12) $$\frac{\tan\delta_{c}}{\tan\delta_{m}}≅\;(1-V_{f})(1+A)$$

where $V_{f}$ is the volume fraction of the filler phase, tan*δ*_c_ the loss tangent of NR–CNF composite and tan*δ*_m_ the loss tangent of pure NR.

## 3. Results and Discussion

### 3.1. Chemical Composition of CNF

The composition of CNF was determined at each stage of chemical treatment and the values are listed in Table 2. The chemical composition of CNF varies from sample to sample. Raw *Cuscuta reflexa* contains 21% hemicellulose, 41% cellulose, and 19% lignin. The percentage of cellulose in CNF was found to be 78%. The cellulose content was improved significantly after each chemical treatment. It was found that the alkali and acid treatments were effective to remove hemicellulose and lignin from raw *Cuscuta reflexa*.

### 3.2. Fourier Transform Infrared Spectroscopy (FTIR)

**Figure 1:**
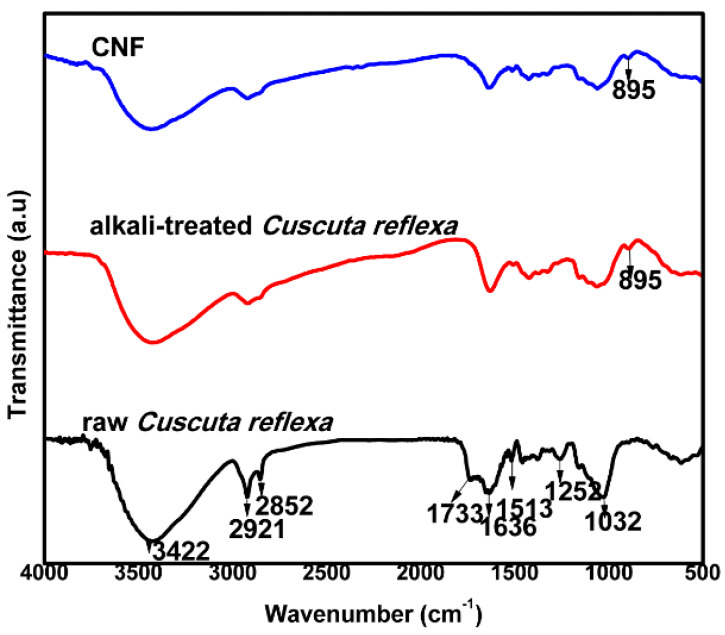
Fourier Transform Infrared Spectroscopy (FTIR) analysis of raw *Cuscuta reflexa*, alkali-treated *Cuscuta reflexa,* and CNF.

The FTIR analysis was conducted to investigate the differences in the chemical structures after the various treatments of *Cuscuta reflexa* fibers. FTIR spectra of raw *Cuscuta reflexa*, alkali-treated *Cuscuta reflexa* and CNF are shown in Figure 1. The bands at 3422 and 2921 cm^−1^ seen in the FTIR spectrum of raw *Cuscuta reflexa* corresponds to the –OH stretching vibration and the –CH group of cellulose respectively [26]. The peak at 2852 cm^−1^ seen in the raw *Cuscuta reflexa* fiber is due to the presence of waxes on its surface [27]. The absence of this peak in alkali-treated and CNF clearly shows the elimination of waxes present in the fiber by chemical treatments. The peak at 1733 cm^−1^ seen in the raw fiber represents the acetyl or uronic ester groups in hemicelluloses [28]. The absence of peaks in the region 1700–1740 cm^−1^ in the alkali-treated *Cuscuta reflexa* fibers shows the effectiveness of chemical treatments in removing hemicellulose. The peak at 1252 cm^−1^ seen in the raw *Cuscuta reflexa* fiber coincides with the C–O out of plane stretching vibration of the aryl group in lignin [29]. The disappearance of this peak in CNF shows the removal of lignin. The peak at 1513 cm^−1^ present in the raw fiber corresponds to C=C aromatic skeletal vibration of lignin [30]. The disappearance of this peak in CNF shows the removal of a significant amount of lignin from the raw fiber. The peak appearing at 1032 cm^−1^ is due to the C–O–C stretching vibrations of the β-1,4-glycosidic ring linkage between the D-glucose units in cellulose which is more pronounced after the acid hydrolysis. Additionally, a significant intense peak at 890 cm^−1^ is observed after acid treatment. This peak corresponds to β-glycosidic linkages of the glucose ring of cellulose [31]. The absorption bands in the region 890–1200 cm^−1^ indicate the hike of cellulose content with different chemical treatments. Due to the hydrophilic nature, all the fibers in the spectra exhibit signals in the range 1603–1640 cm^−1^ which represent the –OH bending vibration of absorbed water [32].

### 3.3. X-Ray Diffraction (XRD) 

**Figure 2:**
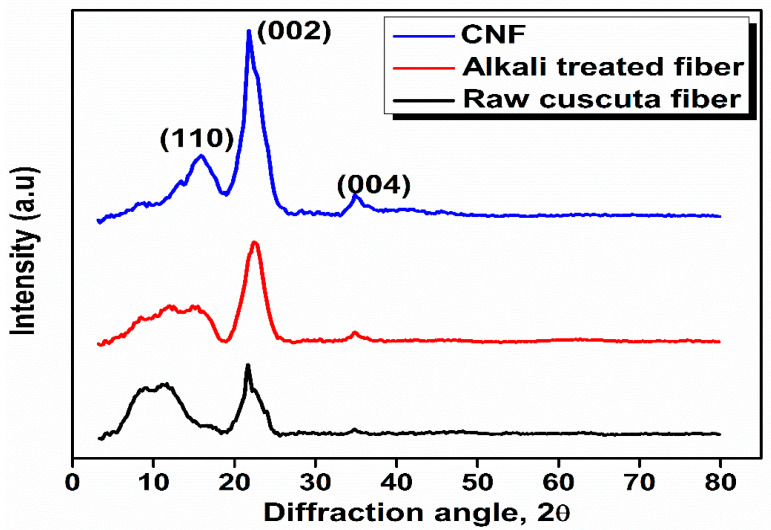
X-Ray Diffraction (XRD) pattern of raw *Cuscuta reflexa*, alkali-treated *Cuscuta reflexa,* and CNF.

The X-ray diffraction pattern of CNF is shown in Figure 2. XRD analysis was conducted to assess the crystallinity index and phase purity of CNF after different chemical treatments. The XRD pattern of CNF shows the characteristic peaks at 2θ = 15.7°, 21.7°, and 37.5° corresponds to the reflections from the plane (110), (002), and (004), respectively [33]. The crystallinity index of raw fiber, alkali-treated fiber, and CNF were found to be 27%, 59%, and 67% respectively. The crystallinity of CNF was determined and compared with raw and alkali-treated fiber in order to examine the potential of the chemical treatment. In the case of raw *Cuscuta reflexa* fiber, the crystalline domains were embedded in the matrix of amorphous components like hemicellulose, lignin, and pectin. There is no doublet in the main peak at 2θ = 22°, which confirms that cellulose in CNF exists as cellulose-I and not cellulose-II [34]. For fibers having high cellulose content, like cotton, flax, etc., two peaks are observed around 2θ = 16° whereas CNF shows only one peak. This phenomenon is related to the presence of cementing materials and amorphous cellulose which cover the two peaks [35]. During the steam explosion process in the presence of an acid, hydronium ions penetrate the amorphous regions of the alkali-treated *Cuscuta reflexa* fiber. This might be responsible for the hydrolytic cleavage of the glycosidic bonds [36]. The removal of amorphous hemicellulose and lignin along with the proper orientation of fibers result in the high value of the crystallinity index of CNF.

### 3.4. Scanning Electron Microscopy (SEM) 

**Figure 3:**
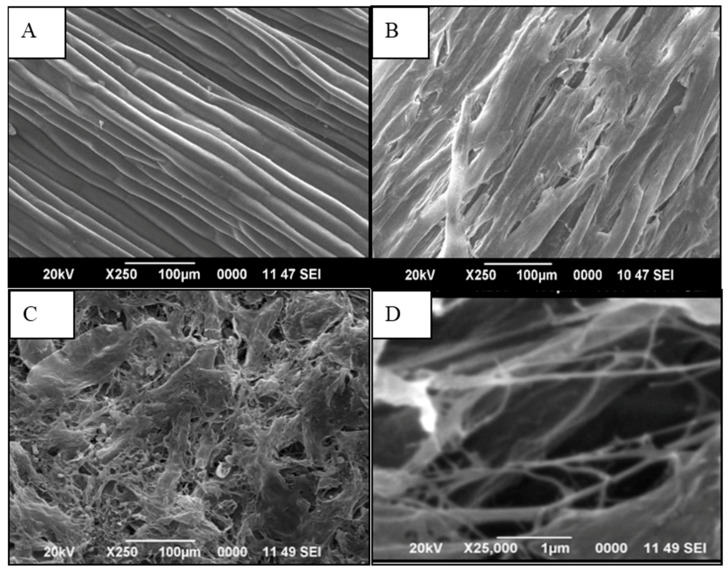
Scanning Electron Microscopy (SEM) images of (**A**) raw *Cuscuta reflexa,* (**B**) alkali-treated *Cuscuta reflexa,* and *(***C**) and (**D**) CNF, under two different magnifications (×250 and ×25,000).

The SEM images of the outer surfaces of *Cuscuta reflexa* after each chemical treatment are shown in Figure 3. Normally raw fibers show an uneven surface with some globular protrusions on its surface. These are silicified stigmata which are minute thickenings. Whereas the SEM image of raw *Cuscuta reflexa* (Figure 3A) shows a smooth surface with a cylindrical morphology which will be a characteristic feature of this parasitic plant. By NaOH treatment, the fibers became thinner. This may be due to the dissolution and leaching out of fatty acids, phenolic compounds, waxes, hemicellulose, and lignin. The removal of these binding materials makes the fibers stiffer and rigid. Figure 3B represents the status of *Cuscuta reflexa* fiber after the mercerization process. The dissolution and leaching out process create a large number of openings on the surface of the fiber. These voids provide better mechanical anchorage and interlocking between the fiber and the polymer matrix [37]. The NaOH treatment increases the number of reactive sites and allows better fiber wetting [38]. The mercerization and steam explosion process make the raw fiber a rough fibrillar structure. The SEM images (Figure 3C,D) show that defibrillation occurs after acid hydrolysis.

### 3.5. Transmission Electron Microscopy (TEM)

**Figure 4:**
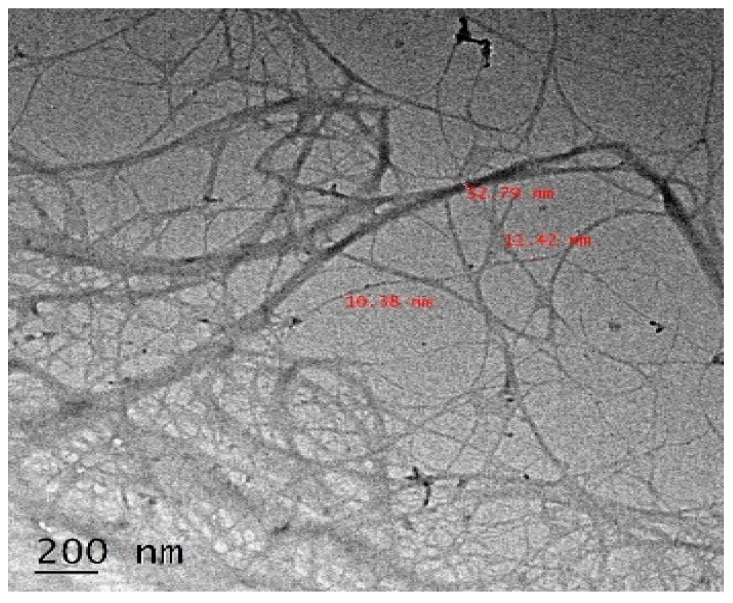
Transmission Electron Microscopy (TEM) image of CNF isolate from *Cuscuta reflexa* plant.

The TEM image of CNF is shown in Figure 4. A well-arranged thread network structure is shown by CNF. This shows the effectiveness of steam explosion method to prepare cellulosic nanofibers. The diameter of CNF varies between 10–30 nm.

### 3.6. Thermogravimetric Analysis of CNF 

**Figure 5:**
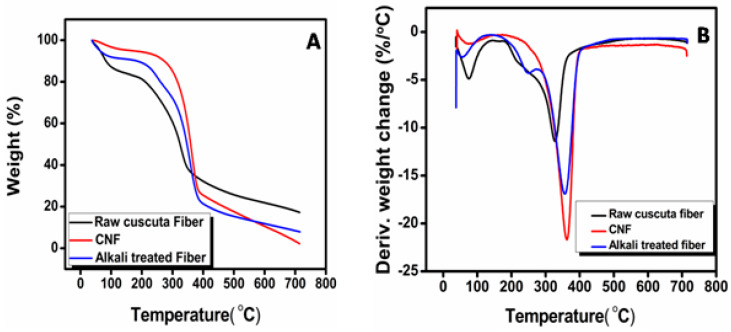
**A**—TG and **B**—DTG curves of CNF.

The thermal decomposition of CNF (Figure 5) happens in two stages. The first stage (250–350 °C) thermal depolymerization of hemicellulose and lignin occurs, along with the breakdown of the crystalline region. The second stage occurs (350–440 °C) due to the thermal oxidative degradation of the char produced. All the samples show an initial weight loss at 100 °C is due to the evaporation of moisture in the fibers. The thermal degradation of raw *Cuscuta reflexa* fiber starts at 260 °C with a maximum rate at 329 °C. The degradation of alkali-treated fiber starts at 304 °C with a maximum rate at 356 °C. The CNF shows a tremendous improvement in its onset degradation temperature. In the case of CNF, degradation starts at 334 °C with a maximum rate at 363 °C. The temperature at which 50% degradation occurs at 322 °C, 346 °C, and 358 °C for raw *Cuscuta reflexa*, alkali-treated *Cuscuta reflexa* and CNF. The better thermal stability of CNF is due to the sequential removal of hemicellulose and lignin [36]. The high degree of crystallinity of CNF offers higher heat resistance and in turn, results in higher thermal stability. The residue at 700 °C of all prepared samples is determined. The values are 17%, 8%, and 3%, respectively, for raw *Cuscuta reflexa* fiber, alkali-treated *Cuscuta reflexa* fiber, and CNF, respectively. The high residue content in raw *Cuscuta reflexa* fiber is due to the presence of ash and lignin. The above results also confirm significant removal of lignin and hemicellulose from raw *Cuscuta reflexa* by alkali and acid treatment. The high crystalline nature along with the absence of a significant amount of lignin and hemicellulose provide better thermal stability to CNF.

### 3.7. Cure Characteristics of NR–CNF Nanocomposites

The cure characteristics of NR–CNF nanocomposites are shown in Table 3. The result shows that the cure time of CNF loaded nanocomposite is higher than neat NR. This is because; CNF has more active surfaces which can absorb the accelerators, thus delaying the cure reaction [39]. Scorch time provides information on processing safety of the rubber compound. Scorch time is higher for CNF reinforced vulcanizate than neat NR. The delayed start of cure reaction in the case of CNF may be attributed to the possible interaction of the fibers with accelerators, making it unavailable for cure reaction. The minimum torque which is an indication of the viscosity of the material is higher for the nanocomposite with 3 phr CNF loading. Differential torque is a measure of the extent of the cross-link formation and the fiber matrix interaction [40]. Differential torque increases up to 3 phr fiber loading and then decreases. The increase in differential torque is due to the restriction of rubber chains by CNF. As the fiber loading increases the entanglement of rubber chains by fiber results in decreased mobility of rubber chains. This results in more stiffness and higher differential torque. Fiber loading at 4 phr cause aggregation of fibers results in poor wetting of polymer chains which will decrease the cross-link density. Therefore, differential torque is less for NR–CNF 4 phr composite. Cure rate index (CRI) is a measure of the speed with which the cure reaction takes place. High CRI is observed in the case of NR–CNF 1 phr composite. After the initial increase, the CRI shows reduction with an increase in filler loading.

The Wolff activity coefficient ($\alpha_{F})$ characterizes the reinforcement effect of the CNF in NR. Higher the value of the Wolff activity coefficient, higher will be the reinforcement effect of the CNF. Consider the following Equation (13):

(13) $$\frac{\Delta M_{x}}{\Delta M_{0}}-1=\alpha_{F}\;\frac{m_{x}}{m_{p}}$$

where $\Delta M_{x}$ is the torque increment of NR–CNF composites in dNm, $\Delta M_{0}$ the torque increment of NR gum in dNm, $m_{x}$ the weight of filler and $m_{p}$ the weight of the polymer.

The Wolff activity coefficient was found to be positive for all the composites prepared. Maximum value of $\alpha_{F}$ was found to be at 2 phr CNF addition owing to the better reinforcing effect of CNF.

### 3.8. Mechanical Properties of NR–CNF Nanocomposites

The mechanical properties of NR–CNF nanocomposites are shown in Table 4. Normally untreated natural fibers decrease the tensile strength of the NR matrix [41]. It is due to the poor adhesion between the hydrophilic fiber and the hydrophobic polymer matrix. When untreated fibers are incorporated into NR, the ability of strain-induced crystallization of NR is badly affected [3]. It was observed that CNF incorporated NR shows improved tensile strength compared to neat NR. The tensile strength of neat NR and NR–CNF composite at 2 phr CNF loading are found to be 20.38 MPa and 22.78 MPa, respectively. Even though the increase is not very high, improvement in the tensile strength of NR without any costly coupling agents is really appreciable. This is the major achievement of the present technique employed. By applying latex stage processing, the CNF will be dispersed in the polymer matrix. The alkaline treatment results in pit formation and striations on the surfaces of the CNF. This will enhance the adhesion between the fiber and the matrix. Fiber dispersion, fiber orientation, fiber concentration, fiber-matrix interaction will be at percolation at 2 phr CNF loading. That will be responsible for the high tensile strength of NR–CNF 2 phr nanocomposite. When the fibers are longitudinally oriented, the fibers were aligned in the direction of strain and the CNF act as effective stress carriers [9]. Apart from this, the formation of Zn/CNF and CNF-CNF networks may also play a key role in enhancing the tensile strength of the composite [42]. The hydrogen bonding between CNF act as a network, which helps to transfer the stress effectively. The tensile results show that the CNF will be stressed under applied load thereby transferring stress from the matrix effectively. The fractographic studies also agree with this argument. At high fiber loading, fiber agglomeration happens. Agglomeration of fibers results in the formation of bundles. Then fiber-fiber interaction over ways fiber-matrix interaction. The fibers will be debonded at the interface creating voids. These voids may act as stress concentration centers that are responsible for the deterioration of tensile strength at 4 phr CNF addition (18.27 MPa). The elongation at break of neat NR and NR–CNF 2 phr composite is 810% and 799%, respectively. Elongation at break of the nanocomposites decreased gradually with increase in the CNF content. This is because of the immobilization of the polymer chains by the CNF network. The incorporation of fiber into the polymer matrix reduces the matrix mobility, resulting in stiffness of the composite [43]. That may be the reason for the decrease in elongation at break. 

That modulus at 300% elongation increases with an increase in the amount of fiber up to 3 phr and then decreases with the further addition of CNF. The modulus at 300% elongation of NR–CNF 1 phr and NR–CNF 3 phr nanocomposites are 2.25 MPa and 2.90 MPa, respectively. The improvement in modulus shows the effective interaction between CNF and NR matrix. The incorporation of fiber into the polymer matrix reduces the mobility of the polymer chains, resulting in stiffness of the composite. The restricted mobility of polymer chains in the vicinity of cross-linked Zn/CNF and CNF-CNF 3D-networks is responsible for the improvement in the modulus. At higher CNF loading the modulus is decreasing, because of the agglomeration of nanofibers and less cross-link density. The tear strength is higher for NR–CNF 2 phr composite (40.77 N/mm) compared to others. The NR–CNF 2 phr nanocomposite shows an improvement in tear strength by 23% compared to neat NR (33.12 N/mm). The increase in tear strength along the longitudinal direction is due to the obstruction created to the tear path by CNF [39]. At 2 phr CNF loading; the nanofibers can effectively resist the crack propagation because of its homogeneous dispersion and proper orientation. The lower value of the tear strength at 4 phr fiber loading (35.49 N/mm) is because of the agglomeration of nanofibers [3].

Hardness is a measure of the modulus of elasticity at low strain. The hardness of NR–CNF nanocomposites increases with increase in CNF loading. The Shore A hardness values of NR gum and NR–CNF 4 phr composite are 37 and 42, respectively. When fiber concentration increases, the stiffness of the rubber composites increases. This is the reason for the improvement in hardness of NR–CNF nanocomposites compared to neat NR. The abrasion resistance index of neat NR is found to be 77%. The abrasion resistance index is maximum for NR–CNF 2 phr composite (80%). The effective interaction between fiber and the matrix results in higher abrasion resistance to the composite [44]. Compression set values increases with increase in CNF loading. When closely packed fibers are compressed in the direction of their alignment, buckling of fibers can happen normally [45]. That might be the reason for the increase in compression set values. The mercerization and latex stage processing increases the interaction between the fiber and the matrix. So, the extent of buckling decreases, which may be the reason for the low value of compression set at a lower loading of CNF. The low compression set value is an indication of low residual deformation happens to the composite. Incorporation of CNF restrains the matrix and increases the elastic nature of NR. The energy dissipated at the fiber matrix interface will be less because of uniform dispersion and proper orientation of nanofibers. This results in an increase in rebound resilience. NR–CNF 2 phr nanocomposite shows the highest resilience.

### 3.9. Swelling Studies of NR–CNF Nanocomposites

**Figure 6:**
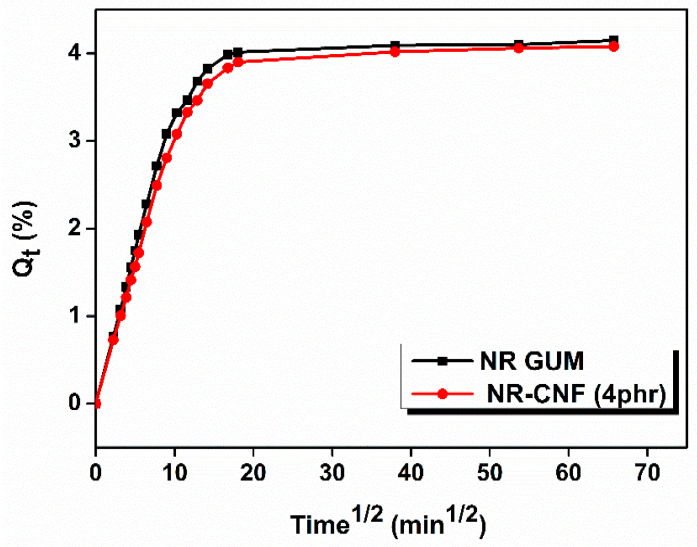
*Q*_t_ vs. *t*^1/2^ plot of NR gum and NR–CNF 4 phr nanocomposites.

Swelling studies of NR–CNF composites were conducted to understand the matrix-fiber interaction. The swelling behavior of the prepared nanocomposites is shown in Figure 6. From the swelling analysis, it is clear that by the addition of CNF into NR matrix, the swelling index decreases to a minimum at 2 phr CNF addition and then increases. The uniform dispersion, proper orientation and better interaction of CNF with NR result in the low value of swelling index at 2 phr CNF addition. The polarity, tortuosity and the three –dimensional networks of CNF might be responsible for the restricted entry of toluene into the rubber matrix. The cross-link density has reached a maximum value at 2 phr CNF addition. The uniform dispersion and the tangling effect of CNF restrict the anisotropic swelling of the composites. The robust composite structure created by the CNF-Zinc complex network may also hinder the entry of solvent molecules to the matrix that will decrease the swelling index of the material [46,47]. Increase in the swelling index at higher CNF loading is because of agglomeration of nanofibers. The elastic Gibb’s free energy (∆G) and change in the conformational entropy (∆S) of different samples upon swelling were also investigated. The value of ∆G, which is a measure of elasticity of the material, is more negative at 2 phr CNF addition, shows better elasticity to that composite compared to others [48]. The uniform dispersion of CNF within the rubber matrix is the main reason for the high value of conformational entropy (∆S) at 2 phr CNF addition. The sorption data is summarized in Table 5.

### 3.10. Fractographic Studies of NR–CNF Nanocomposites

**Figure 7:**
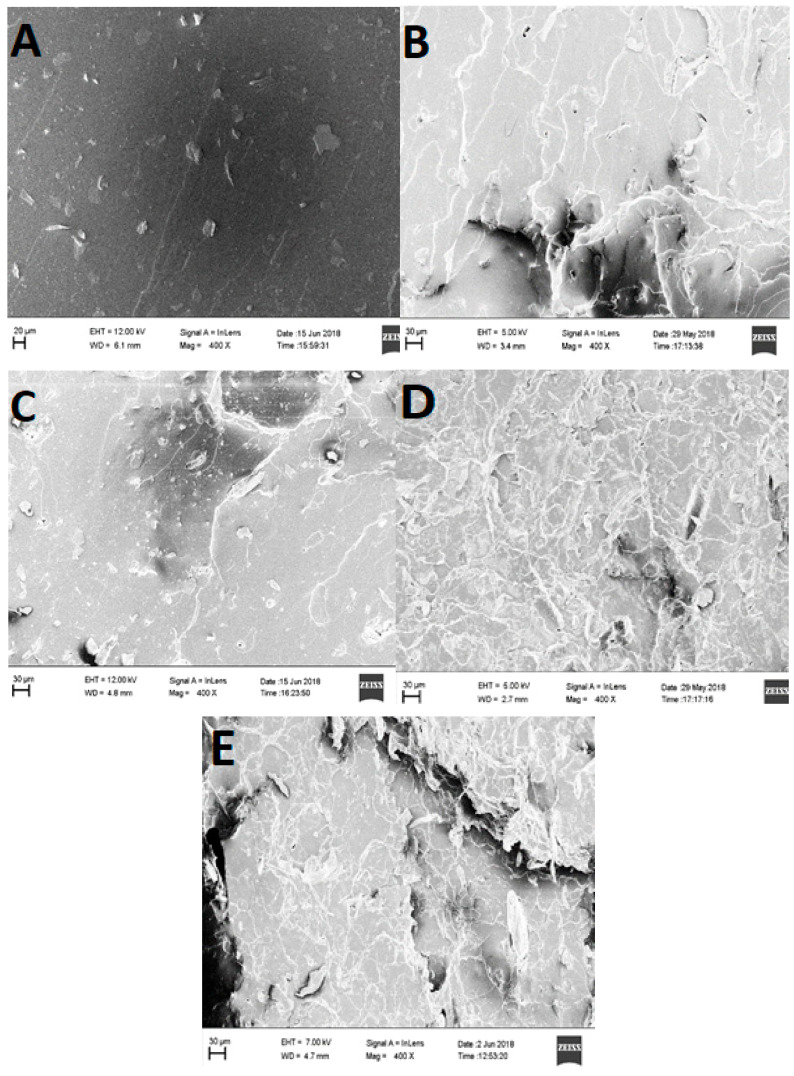
FESEM images of tensile fractured samples of vulcanizates. (**A**) NR gum, (**B**) NR–CNF 1 phr, (**C**) NR–CNF 2 phr, (**D**) NR–CNF 3 phr, and (**E**) NR–CNF 4 phr.

The fracture of CNF reinforced composites occurs in two modes, either by breakage of CNF leading to failure or pullout of several fibers from the matrix. The fractured surface of NR gum seems to be smooth (Figure 7A). The flat surface with ragged zones shows the typical fracture mechanism of elastomers. As CNF loading increases the surface became irregular and rough. This shows plastic deformation and a fracture mechanism transition. The craters, undulations, and roughness of NR–CNF nanocomposites (Figure 7B–D) indicate that the fracture process is relatively slower [49]. Effective matrix- fiber interactions are observed at 2 and 3 phr CNF loading, represented in Figure 7C,D. The SEM image of the tensile fractured sample of NR–CNF 4 phr composite (Figure 7E) shows voids and fiber pullouts. These voids act as centers for crack propagation, which will ultimately result in inferior mechanical properties [50].

### 3.11. Thermogravimetric Analysis of NR–CNF Composites

The thermogravimetric analysis data are summarized in Table 6. The main degradation occurs between 250 °C and 400 °C in all the NR–CNF nanocomposites is due to the chain scission and cross-linking bonds breakage. The onset degradation temperature of NR Gum, NR–CNF 1 phr, and NR–CNF 2 phr are 300, 316, and 304 °C, respectively. This shows the effectiveness of CNF in delaying the thermal degradation of the rubber matrix. The increase in the onset degradation temperature is due to the removal of low thermally stable hemicellulose from the raw fiber. The decomposition of CNF results in the formation of volatiles and char. These residues act as a protective barrier to both mass and energy from the burning surface to the attached polymeric chains [42]. The decreased mobility of NR phase in the proximity of CNF results in slower diffusion of degradation products from the material [51]. Zn-cellulose complex and percolation system play a significant role in the better thermal stability at lower NR–CNF addition [47]. The reduction in thermal stability at higher fiber loading is due to the agglomeration of the fibers. The thermal analysis shows that there is no remarkable improvement in *T*_50_ (the temperature at which 50% degradation occurs) of the prepared nanocomposites. The residue of nanocomposites at 500 °C is increasing with the increase in CNF loading. The high residue indicates the increase in crystalline cellulose in CNF. 

### 3.12. Dynamic Mechanical Analysis

**Figure 8:**
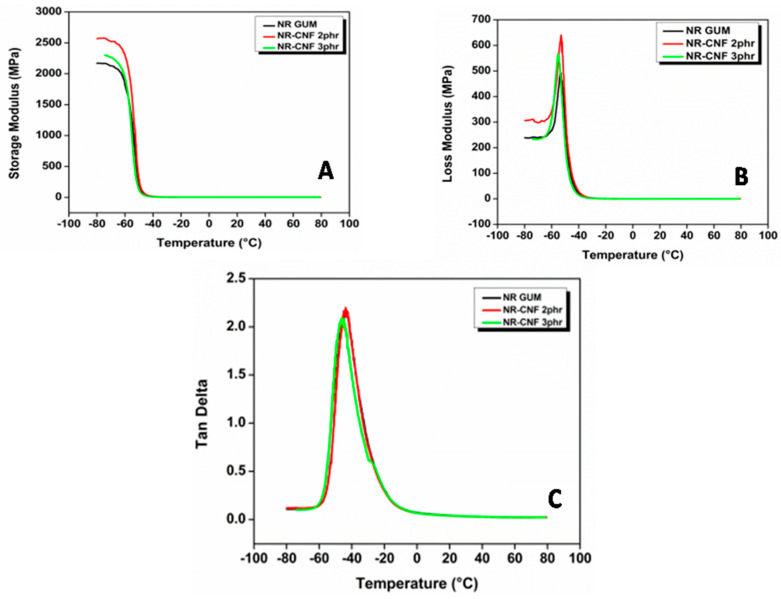
(**A**) Variation of storage modulus with temperature, (**B**) variation of loss modulus with temperature and (**C**) variation of loss tangent with temperature of NR–CNF nanocomposites.

Figure 8A shows the variation of storage modulus of different NR–CNF composites with temperature. Dynamic mechanical analysis data of NR-CNF composites are summarized in Table 7. Storage modulus measures the maximum energy stored in NR–CNF composites during one cycle of oscillation. The DMA analysis shows that the storage modulus in the glassy region is highest for NR–CNF 2 phr composite. The high value of the storage modulus is due to the homogenous dispersion of CNF in NR. The value of the storage modulus is an indication of hardness and crosslink density of composites [13]. The high cross-links between NR and CNF provide more stiffness to NR–CNF 2 phr composite which results in its high storage modulus. By mercerization process, the surface of *Cuscuta reflexa* fibers becomes rough, owing to the strong interfacial bonding between CNF and NR. The increased surface area of CNF provides improved wetting by polymer chains which also may be the reason for the improvement in storage modulus. At around −60 °C, a sharp decrease in storage modulus was observed for all NR–CNF composites because of the energy dissipation phenomenon. At higher temperature (*T* > glass transition temperature) the storage modulus was found to be almost constant and a plateau was achieved. The storage modulus at 25 °C of NR-gum, NR–CNF 2 phr, and NR–CNF 3 phr composites are 1.56, 2.22, and 1.87 MPa, respectively. The crystalline domains of CNF would behave as physical-crosslinks for the rubber chains that would increase the storage modulus significantly. Similar results were observed when crab shell chitin nanowhiskers are introduced as filler in NR [52]. The increase in rubbery modulus at 2 phr CNF addition is evidence of high degree crystallinity of the material. The effectiveness of CNF on storage moduli of the composites is also determined from the coefficient C [53]. The measured values of storage modulus (E′) −50 °C and 50 °C were used as glassy and rubbery storage modulus, respectively. The low value of C shows the higher effectiveness of CNF on storage modulus. The C value of NR–CNF 2 phr and NR–CNF 3 phr are 0.83 and 0.88, respectively. The effectiveness of CNF on storage moduli is maximum at 2 phr CNF addition. The low value of C for NR–CNF 2 phr composite indicates better matrix–fiber interactions.

Figure 8B shows the variation of loss modulus (E″) with the temperature of NR gum and NR–CNF nanocomposites. The loss modulus indicates the energy lost to friction in NR–CNF nanocomposites. From the maxima of the loss modulus curve (E″) versus temperature, glass transition temperature (*T*_g_) of the composites was determined. The *T*_g_ of NR gum, NR–CNF 2 phr, and NR–CNF 3 phr are −53.29 °C, −53.11 °C, and −55.20 °C, respectively. The DMA result shows that *T*_g_ values are almost unaffected by the addition of CNF into NR matrix. The slight, positive shift in the glass transition temperature of NR–CNF 2 phr composite indicates the better interaction of CNF with natural rubber.

The CNF shows the tendency to agglomerate and the entrapped rubber segments in the aggregate could not participate in rubber chain relaxation process. That might be the reason for the decrease in glass transition temperature at higher CNF loading. This is also in consistent with rubber shell theory. The maximum loss modulus of NR gum is less than that of the composites. The increase in E″ of nanocomposites is due to the energy dissipation which arises from a breakdown of CNF transient networks and loss of trapped rubber chains on the filler surface during dynamic tests [54].

Figure 8C shows the variation of loss tangent (tan δ) of NR Gum and NR–CNF composites with temperature. tanδ is the ratio of dissipated energies and storage energies. It is a parameter to determine the internal friction [55]. Higher the value of loss tangent higher will be the internal friction the system suffers and more heat would be developed in the system. The magnitude of the maximum tan δ value of NR Gum, NR–CNF 2 phr, and NR–CNF 3 phr are 2.17, 2.20, and 2.08, respectively. The decrease in tan δ peak height in NR–CNF 3 phr composite is clear evidence of hindering of rubber molecular chains by CNF [56]. The higher degree of fibrillation of CNF results in mechanical interlocking with NR matrix. The homogenous dispersion of CNF in the matrix results in the confinement of NR chains which results in a low value of tanδ. The improvement in the interfacial reinforcement of NR–CNF nanocomposite is evident from the lowering of tanδ peak. Thus we can conclude that CNF reinforced composites shows better dynamic properties than NR Gum and these nanofibers decreases the damping of the composites. *T*_g_ with respect to maximum tanδ of NR Gum, NR–CNF 2 phr and NR–CNF 3 phr are −44.45, −43.77, and −46.11 °C, respectively. The marginal improvement in *T*_g_ of NR–CNF 2 phr composite shows the reinforcing action of CNF. The volume fraction of immobilized polymer chain (*C*_v_) of NR–CNF 2 phr and NR–CNF 3 phr composite are found to be 0.0183 and 0.0009. The homogenous dispersion of CNF in NR provides better interfacial interaction between the fiber and the polymer chains. This might be the reason for the high value of the volume fraction of the constrained region at 2 phr CNF loading. Dileep et al. reported similar improvements in *C*_v_ when silica fume is used as a filler in NR [57].

The adhesion parameter (A) of NR–CNF 2 phr and NR–CNF 3 phr nanocomposite are found to be −0.0011 and 0.0375. Either, NR–CNF 2 phr composite has a high value of *C*_v_ and low value of A. Lower the value of A, higher will be the interphase addition and enhanced interaction between CNF and NR [13].

## 4. Conclusions

In summary, cellulosic nanofibers (CNF) were extracted from *Cuscuta reflexa*, a highly dangerous parasitic plant. The nanofibers were characterized using FTIR, XRD, SEM, TEM, and TGA. The FTIR spectra confirm the removal of non-cellulosic materials during chemical treatments. XRD analysis shows a high crystallinity index of CNF (67%). SEM images underline the fibrous morphology of CNF. TEM images show that the diameter of CNF is in between 10 and 30 nm. The TGA analysis shows that the thermal stability of CNF is higher than that of raw *Cuscuta reflexa* fiber. NR–CNF green nanocomposites were prepared by incorporating a masterbatch of CNF in dry rubber compounding. Better cure rate index was found to be at 1 phr CNF addition. The tensile strength and tear strength were found to be higher for NR–CNF 2 phr nanocomposite. The swelling index was also found to be minimum for NR–CNF 2 phr nanocomposite. The onset degradation temperature of NR can be improved by the addition of CNF, whereas *T*_50_ remained unaffected. The DMA analysis shows that there is not much difference in the glass transition temperature of neat NR and NR–CNF composites. The DMA result also showed that the value of *C*_v_ is maximum for NR–CNF 2 phr composite. The fractographic studies show the uniform distribution of CNF in NR, resulting in higher reinforcement. Based on presented results, it can be concluded that CNF seems to be a promising green filler for developing eco-friendly natural rubber based composites.

## Figures and Tables

**Table 1:** Formulation and coding of natural rubber (NR)–cellulose nanofiber (CNF) composites.

Sample Code	Solid NR (phr)	Masterbatch		ZnO (phr)	Stearic Acid (phr)	Nonox SP (phr)	CBS (phr)	Sulphur (phr)
		NR (phr)	CNF (phr)					
NR-gum	50	50	-	5	2	1	0.6	2.5
NR–CNF 1 phr	50	50	1	5	2	1	0.6	2.5
NR–CNF 2 phr	50	50	2	5	2	1	0.6	2.5
NR–CNF 3 phr	50	50	3	5	2	1	0.6	2.5
NR–CNF 4 phr	50	50	4	5	2	1	0.6	2.5

**Table 2:** Chemical composition analysis of cellulose nanofiber (CNF) at each stage of treatment.

Samples	Hemicellulose (%)	Cellulose (%)	Lignin (%)
raw *Cuscuta reflexa*	21 ± 4	41 ± 5	19 ± 3
alkali-treated *Cuscuta reflexa*	14 ± 4	74 ± 5	12 ± 4
CNF	12 ± 5	78 ± 5	10 ± 3

**Table 3:** Curing parameters and Wolff activity coefficient determined for NR–CNF composites.

Properties	NR-Gum	NR–CNF 1 phr	NR–CNF 2 phr	NR–CNF 3 phr	NR–CNF 4 phr
Scorch time, *t*_s2_(min)	2.49	3.27	3.26	3.25	3.07
Optimum cure time, *t*_90_(min)	7.42	8.16	8.16	8.42	8.19
Cure rate index (min^−1^)	20.28	20.44	20.40	19.34	19.76
Minimum torque(*M*_L,_ dNm)	0.15	0.20	0.20	0.25	0.16
Maximum torque(*M*_H,_ dNm)	6.71	6.96	7.24	7.47	7.31
Differential torque, *M*_H_–*M*_L_ (dNm)	6.56	6.76	7.04	7.22	7.15
Wolff activity coefficient	-	3.04	3.65	3.35	2.24

**Table 4:** Mechanical properties of NR–CNF nanocomposites.

Properties	NR-Gum	NR–CNF 1 phr	NR–CNF 2 phr	NR–CNF 3 phr	NR–CNF 4 phr
Tensile strength (MPa)	20.38 ± 0.44	20.50 ± 0.5	22.78 ± 0.52	22.28 ± 0.42	18.27 ± 0.38
Modulus at 300% elongation (MPa)	2.11 ± 0.04	2.25 ± 0.02	2.70 ± 0.05	2.90 ± 0.1	2.40 ± 0.12
Elongation at break (%)	810 ± 0	819 ± 9	799 ± 11	778 ± 10	763 ± 12
Tear strength (N/mm)	33.12 ± 2.51	34.04 ± 1.12	40.77 ± 1.11	37.58 ± 1.2	35.49 ± 1.09
Hardness (Shore A)	37 ± 1	38 ± 1	41 ± 1	41 ± 1	42 ± 1
Compression set (%)	3.21 ± 0.25	3.27 ± 0.23	3.27 ± 0.26	3.55 ± 0.28	3.85 ± 0.21
Rebound resilience (%)	77 ± 2	78 ± 3	79 ± 2	77 ± 4	77 ± 3
Abrasion resistance index, ARI (%)	77 ± 3	78 ± 1	80 ± 1	79 ± 1	78 ± 2

**Table 5:** Sorption parameters of NR–CNF nanocomposites.

Properties	NR-Gum	NR- CNF 1 phr	NR–CNF 2 phr	NR–CNF 3 phr	NR–CNF 4 phr
Swelling index (%)	382	380	368	370	375
Cross-link density (×10^−5^ mol/g)	3.11	5.26	5.72	5.37	5.36
Molar mass between cross-links (g/mol)	16077	9505	8741	9310	9328
ΔG (J/mol)	−7.73	−8.23	−9.01	−8.54	−8.43
ΔS (×10^2^ J/mol K)	2.59	2.76	3.02	2.86	2.82

**Table 6:** Thermal stability of NR–CNF nanocomposites.

Samples	Degradation Temperature (°C)				Residue at 500 °C (%)	Maximum Degradation Temperature (*T*_max_, °C)
	*T*_on_	*T*_5_	*T*_50_	*T*_90_		
NR gum	300	289	387	439	8.53	379
NR–CNF 1 phr	316	304	388	475	10.08	381
NR–CNF 2 phr	304	293	386	491	10.50	378
NR–CNF 3 phr	302	292	384	504	11.20	378
NR–CNF 4 phr	293	283	372	526	12.67	364

**Table 7:** Dynamic mechanical analysis data of NR gum and NR–CNF nanocomposites.

Properties	NR- gum	NR–CNF 2 phr	NR–CNF 3 phr
*T*_g_(according to tanδ max) (°C)	−43.74	−43.77	−46.11
*T*_g_ (according to *E*” max) (°C)	−53.29	−53.11	−55.20
tanδ at 25 °C	0.0369	0.0361	0.0368
Storage Modulus (MPa)	1.56	2.22	1.87
Coefficient *C*	1	0.83	0.88
Volume fraction of immobilized polymer chain, (*C*_v_)	0	0.0183	0.0009
Adhesion factor, *A*	0	−0.0011	0.0375
